# Academic Stress and Emotional Well-Being in United States College Students Following Onset of the COVID-19 Pandemic

**DOI:** 10.3389/fpsyg.2021.628787

**Published:** 2021-03-17

**Authors:** Alison Clabaugh, Juan F. Duque, Logan J. Fields

**Affiliations:** Department of Psychology, Arcadia University, Glenside, PA, United States

**Keywords:** college students, COVID-19, emotional well-being, higher education, pandemic, stress

## Abstract

COVID-19 has resulted in extraordinary disruptions to the higher education landscape. Here, we provide a brief report on 295 students’ academic perceptions and emotional well-being in late May 2020. Students reported the high levels of uncertainty regarding their academic futures as well as significant levels of stress and difficulty coping with COVID-19 disruptions. These outcomes were related to the higher levels of neuroticism and an external locus of control. Female students reported worse emotional well-being compared to males, and the students of color reported the significantly higher levels of stress and uncertainty regarding their academic futures compared to White students. These results suggest that some students may be at particular risk for academic stress and poor emotional well-being due to the pandemic and highlight the urgent need for intervention and prevention strategies.

## Introduction

In response to the COVID-19 pandemic, over 1,000 colleges and universities in the United States closed their doors in March 2020. Millions of students were forced to finish the semester *via* remote learning, resulting in extraordinary disruptions to higher education in the United States (Goldstein, 2020). Although COVID-19 poses a low risk to the health and mortality of college-aged students (Centers for Disease Control and Prevention, 2020), the pandemic has likely resulted in stark uncertainty and distress in this population.

One particular area of concern for students in higher education is academic stress relating to their ability to succeed in this new environment. While enrollment in online courses has increased over the past several years, the majority of students remain unfamiliar with remote learning. A recent report indicates that prior to COVID-19, only 35% of United States college students had taken one or more courses online (D’Amato, 2020). This concerning given that one of the best predictors of academic success in an online format is prior online course experience (Hachey et al., 2012). This lack of experience may be compounded by challenging home conditions, including loss of access to academic resources (e.g., computers and internet connectivity) and distractions in the home learning environment. Indeed, the initial research shows that at-home distractions (including disruptions from other family members and additional responsibilities) are a significant challenge for college students learning from home during COVID-19 (Son et al., 2020). Taken together, these factors are likely to lead to significant academic stress and uncertainty.

Aside from dealing with stressors related to a potentially unfamiliar online learning environment, students are also coping with the emotional impact of COVID-19. Much of the initial research on the mental health consequences of COVID-19 comes from areas hardest hit at the beginning of the pandemic including countries in Asia and Europe. This research shows that COVID-19 and its associated disruptions have resulted in significant increases in stress, anxiety, depression, and suicidality in college students (Husky et al., 2020; Li et al., 2020; Luo et al., 2020; Patsali et al., 2020). More recent investigations in the United States indicate that college students show a similar pattern in mental health and well-being to those from other regions of the world coping with COVID-19 (e.g., Luo et al., 2020; Son et al., 2020). Unfortunately, studies from the United States addressing these phenomena thus far have focused on students from single institutions and have under-explored gender and ethnic differences in COVID-19 related mental health issues. These are crucial to investigate, particularly because men and ethnic minorities are more likely to experience negative health outcomes after exposure to COVID-19 (Griffith, 2020), while women and ethnic minorities are more likely to suffer negative occupational and mental health consequences due to the pandemic (Adams-Prassl et al., 2020; Alonzi et al., 2020; NAACP, 2020). These differences are crucial to investigate, particularly, because the initial research suggests that women and ethnic minorities are more likely to suffer adverse changes in their emotional well-being due to the pandemic (Adams-Prassl et al., 2020; Alonzi et al., 2020; Rothman et al., 2020; Smith et al., 2020; Thibaut and van Wijngaarden-Cremers, 2020). For example, using a large, the geographically representative sample of United States adults, Adams-Prassl et al. (2020) documented a significant decrease in mental health as a result of initial COVID-19 stay-at-home orders. Of note, this decrease was entirely driven by worsening mental health in females. Similarly, research on ethnic minority populations suggests that the pandemic is likely to exacerbate pre-existing mental health disparities due to significant rates of COVID-19 infection in these communities as well as quarantine-related impediments to mental health care (Rothman et al., 2020; Smith et al., 2020). Thus, many students (women and minority populations in particular) are likely facing challenges to their well-being during the pandemic.

Emotional well-being during the times of turmoil depends on factors at both the individual and societal level. Thus far, research on emotional well-being during COVID-19 has focused on societal-level factors including response to situational stressors (e.g., infection fears, constraints on physical movement, limited social contact, and sudden lifestyle changes). What remains under-explored is how the effects of these stressors may vary based on individual differences such as personality traits. Neuroticism, for example, has profound implications for mental and physical health (e.g., Lahey, 2009; Widiger and Oltmanns, 2017). Research shows that individuals who are high in neuroticism are at increased risk for negative physical health outcomes and the various forms of psychopathology including anxiety and mood disorders (see Tackett and Lahey, 2017 for a review). For example, a recent investigation in Germany found that individuals with higher neuroticism attended to and worried about the ongoing COVID-19 pandemic more than those lower on neuroticism (Kroencke et al., 2020). Additionally, locus of control (LoC) has been shown to predict the ability to cope with stressful life experiences (Zeidner, 1993; Lefcourt, 2013). During the SARS pandemic of 2003, having a more external LoC was associated with the development of PTSD following a SARS infection (Mak et al., 2010). Thus, it is likely that these individual differences also influence students’ well-being during the COVID-19 pandemic.

The goals of the current study were 2-fold. First, in an effort to capture the impacts of COVID-19 on the higher-education landscape, we explored academic perceptions, emotional well-being, and individual differences among United States college students during the beginning stages of the pandemic in April and May 2020. As part of this exploration, we also assessed students’ COVID-19 perceptions and behaviors and examined relationships between all variables of interest. Second, given that female and ethnic minority students are disproportionately likely to suffer negative occupational and mental health consequences related to the pandemic, we investigated gender and ethnic differences. In light of the recency of the pandemic, this study was exploratory and descriptive in nature. Such studies are a necessary first step toward understanding pandemic-related well-being and can inform later investigations that are more targeted and theory-driven.

## Materials and Methods

### Procedure

A Qualtrics survey was distributed to students at the Arcadia University (Glenside, PA) *via* an online psychology major’s community as well as through various department chairs. Outside of Arcadia, the link was distributed to psychology department chairs at institutions near Philadelphia, PA, including in OH, NJ, NY, DE, and Washington D.C. and was posted to an online teaching Listserv, so that members could distribute the survey link to their students at their own discretion. Participation was voluntary and not compensated.

The survey took ~10 min to complete. Multiple measures were administered; some of which were for another study and are not further reported here. Measures for this study were administered in the following order: demographics, questions assessing COVID-19 perceptions and behaviors, academic perceptions, locus of control, perceptions of stress within the past month, and neuroticism. The IRB at Arcadia University approved all procedures.

### Participants

Three-hundred and 45 individuals started the survey. Two were removed for not meeting the inclusion criteria (full- or part-time undergraduate at least 18 years of age). Fifty participants were removed for incomplete data leaving a final sample of 295 participants (see Table 1). Eighty-five percent of respondents were from colleges in the Northeast United States Notably, no student indicated that they had tested positive for COVID-19 (four did not answer), and 97% reported that no one in their immediate family had tested positive (four reported yes, and five did not answer).

**Table 1:** Student self-reported demographics.

Demographics		
Factor		Sample size (*n*)/% of total
**Gender**		
	Female	237/80.3
	Male	51/17.3
	Other	7/2.4
**Ethnicity**		
	White	212/71.9
	Hispanic/LatinX	30/10.2
	Black	26/8.8
	Other	14/4.7
	Asian	12/4.1
**Age**		
	18–20	168/56.9
	21–23	102/34.7
	24–26	10/3.5
	27+	9/2.8

Of note, these data were collected from mid-April to May 8, when the survey was made inactive. During this time much of the United States was undergoing extensive stay at home orders, in varying forms, generally allowing only essential businesses to remain open (Moreland, 2020). Additionally, at the time of data collection, wearing masks in public was not uniformly recommended and was therefore not assessed.

### Measures

#### Academic Perceptions

Participants responded to three questions assessing academic concerns related to COVID-19: “To what extent do you think your academic future is at risk due to COVID-19?,” “What is the likelihood that you would reduce (or withdraw) from your courses in the Fall of 2020 if classes were still completely or predominantly online due to COVID-19?,” and “To what extent is distraction an issue in your current environment?.” Additionally, participants rated their level of agreement with the statement, “Transitioning to a completely online education is the correct response for schools and universities to take in response to COVID-19.”

#### Emotional Well-Being

Participants responded to a four-item Perceived Stress Scale (Cohen et al., 1983) to assess the degree to which individuals perceive events in their lives within the past month as stressful on a 1–5 scale, ranging from “never” to “very often” (*α* = 0.78). Participants also responded to a single item, “Compared to those around you (e.g., family, friends, and co-workers), how well do you feel you are coping with disruptions in your life caused by COVID-19,” on a 1–5 scale, ranging from “not well at all” to “extremely well.”

#### Personality

Participants responded to a nine-item brief LoC scale (Sapp and Harrod, 1993) to assess the extent to which individuals perceive control over their own lives and the events around them on a 1–5 scale, ranging from “strongly disagree” to “strongly agree.” Higher scores indicate a more internal LoC (*α* = 79). Participants also responded to an five-item Neuroticism subscale of the Big Five Inventory (John and Srivastava, 1999) to assess the extent to which a person is prone to worry and emotional instability on a 1–5 scale, ranging from “strongly disagree” to “strongly agree” (*α* = 0.84).

#### COVID-19 Perceptions and Behaviors

Participants responded to three questions assessing COVID-19 perceptions and behaviors, adapted from Wise et al. (2020): “How serious do you believe COVID-19 is?,” “How do you think you will be affected if you personally catch the virus?,” and “How often are you completing risk management behaviors?”

## Results

### Descriptives

#### Academic Perceptions and Emotional Well-Being

**Figure 1:**
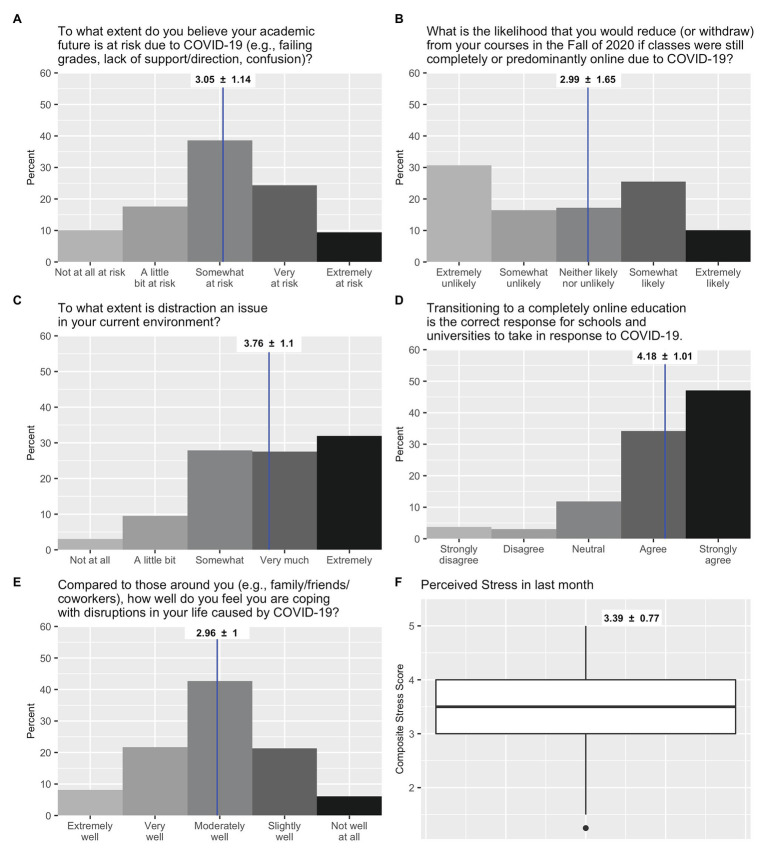
Percent response frequencies for COVID-19 academic perceptions **(A–E)** and boxplot for Perceived Stress Scale **(F)**. Means and standard deviations are listed at the top of each graph.

One-third of students (33%) felt their academic future was “very” or “extremely” at risk due to COVID-19 (Figure 1A), and 32% reported being “somewhat” or “extremely” likely to reduce or withdraw from classes in the Fall of 2020 if classes are completely or predominantly online (Figure 1B). Sixty percent reported that distraction was “very much” or “extremely” an issue in their current environment (Figure 1C). Nonetheless, 81% of students “agreed” or “strongly agreed” that transitioning to an online education in the spring of 2020 was the correct response to take (Figure 1D).

Regarding emotional well-being, 30% of students reported that they were coping “slightly well” or “not well at all” with COVID-19 disruptions (Figure 1E) and reported stress levels were significantly above the scale mid-point of 2.5 (*M* = 3.39, *SD* = 0.77, *t*(286) = 19.46, *p* < 0.01, *d* = 1.16; Figure 1F).

#### COVID-19 Perceptions and Behaviors

**Figure 2:**
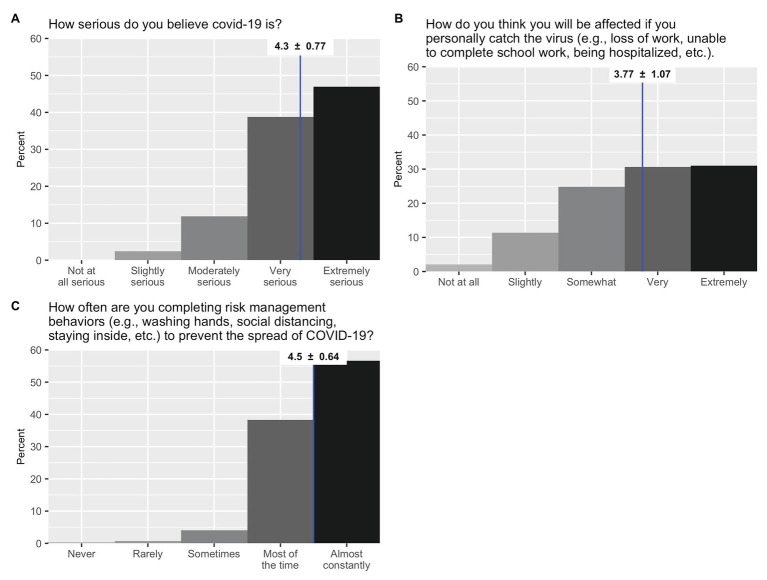
Percent response frequencies for questions related to COVID-19. Means and standard deviations are listed at the top of each graph.

Regarding COVID-19, 86% of students characterized the virus as “very” or “extremely serious” (Figure 2A), and 62% reported they would be “very” or “extremely” affected if they were to catch the virus (Figure 2B). Nearly all students (95%) reported engaging in risk management behaviors either “most of the time” or “almost constantly” (Figure 2C).

### Aim1: Correlational Analyses

We first sought to assess the relationship between personality variables (neuroticism; *M* = 3.35, SD = 0.77; LoC; *M* = 3.46, SD = 0.64), academic perceptions, and emotional well-being (see Table 2). Both neuroticism and LoC showed similar relationships with academic concerns. Specifically, the higher levels of neuroticism and a more external LoC were associated with perceptions of academic future being at greater risk, higher likelihood of reducing or withdrawing from online courses in the fall, and the higher reported levels of distraction in the home learning environment. Agreement that the transition to online education was the “correct” response to the pandemic was not related to any personality variables but was significantly related to lower risk perception for academic future, lower likelihood of reducing or withdrawing from online classes in the fall, lower levels of distraction in the home learning environment, and better coping with COVID-19 disruptions. Thus, students coping well with academic concerns and COVID-19 tended to agree that transitioning online was the correct choice.

**Table 2:** Bivariate correlations between academic perceptions, emotional well-being, personality measures, and COVID-19 perceptions and behaviors.

Abbreviated Variables	1	2	3	4	5	6	7	8	9	10
1. Academic: Belief future at risk^A^	-									
2. Academic: Likelihood reduce or withdraw^B^	0.3^**^	-								
3. Academic: Extent distraction an issue^C^	0.28^**^	0.25^**^	-							
4. Academic: Agree online transition correct choice^D^	−0.26^**^	−0.24^**^	−0.23^**^	-						
5. Emotional well-being: Stress^E^	0.51^**^	0.28^**^	0.37^**^	−0.12	-					
6. Emotional well-being: Coping	−0.31^**^	−0.19	−0.36^**^	0.28^**^	−0.56^**^	-				
7. Personality: Locus of Control	−0.29^**^	−0.16	−0.22^**^	0.15	−0.42^**^	0.33^**^	-			
8. Personality: Neuroticism	0.23^**^	0.19	0.25^**^		0.5^**^	−0.41^**^	−0.27^**^			
9. Perception: How serious is COVID-19^A^				0.14				0.12		
10. Perception: Degree affected if catch^B^	0.16		0.13		0.11	−0.14		0.11	0.27^**^	
11. Behavior: Freq. of Risk management^C^				0.14	0.11			0.15	0.23^**^	

*N* = 280–295. Variables A–F and A–C are shown in Figures 1, 2, respectively. Correlations within −0.1 and 0.1 are not shown.

** Reflects significant correlations below the Bonferonni-corrected alpha (*p* = 0.05/66 = 0.0009).

Regarding emotional well-being, the higher levels of neuroticism and a more external LoC were associated with the higher levels of stress and worse coping. Academic concerns (variables 1–3) were also significantly related to poor emotional well-being (variables 5 and 6). For example, students reporting the higher levels of concern about their academic future reported higher stress and worse coping.

Lastly, we investigated whether students’ COVID-19 perceptions and behaviors were related to their academic perceptions or emotional well-being. Though the perceptions of COVID-19 severity correlated with the degree to which students believed that they would be affected if they were to catch the virus, as well as their frequency of risk management behaviors, none of these COVID-19 questions were related to academic perceptions or emotional well-being (Table 2).

### Aim2: Gender and Ethnic Differences

Our second aim was to investigate gender and ethnic differences in COVID-19 related academic perceptions and emotional well-being (see Table 3). Females reported the significantly higher levels of distraction in their home learning environment compared to males; however, there were no other significant gender differences in academic perceptions. With regard to personality and emotional well-being, females had the higher levels of neuroticism, more perceived stress, and worse coping compared to males. Females also reported the higher levels of perceived COVID-19 severity as well as greater frequency of engaging in risk management behaviors.

**Table 3:** Exploratory gender and ethnic differences.

	Male	Female			
**Measure**	**M (SD)**	**M (SD)**	***t*-value**	***p***	**Cohen’s *d***
Academic: Extent distraction an issue	3.39 (1.18)	3.83 (1.07)	−2.57	0.01	0.39
Emotional well-being: Stress	3.03 (0.82)	3.45 (0.74)	−3.55	<0.01	0.54
Emotional well-being: Coping	3.31 (1.14)	2.90 (0.95)	2.42^A^	0.02	0.39
Personality: Neuroticism	2.86 (0.76)	3.44 (0.73)	−5.02	<0.01	0.78
Perception: How serious is COVID-19	4.02 (0.93)	4.35 (0.72)	−2.78	0.01	0.40
Behavior: Freq. of risk management	4.31 (0.65)	4.54 (0.64)	−2.33	0.02	0.36
	**White**	**SoC**			
Academic: Belief future at risk	2.95 (1.15)	3.31 (1.08)	−2.49	0.01	0.32
Academic: Likelihood reduce or withdraw	2.90 (1.71)	3.23 (1.46)	−1.65^A^	0.10	0.21
Behavior: Freq. of risk management	4.56 (0.55)	4.36 (0.82)	2.00^A^	0.05	0.29
Perception: Degree affected if catch	3.67 (1.07)	4.05 (1.04)	−2.77	<0.01	0.36

Only significant differences are shown.

A Welch’s *t*-test used due to the homogeneity of variance assumption being violated (Levene’s test).

Compared to White students, students of color (SoC: Black, Hispanic/Latinx, Asian, and Other) reported the perceptions of greater risk for their academic future and higher likelihood of reducing or withdrawing from online classes in the fall (although this difference was only marginally significant). Similarly, SoC reported that they would be more severely affected if they were to contract COVID-19 than White students. Somewhat unexpectedly, White students reported significantly more frequent engagement in risk management behaviors (e.g., washing hands) than SoC. There were no significant ethnic differences on stress, coping with COVID-19 disruptions, or either of the personality variables.

## Discussion

In an effort to contribute to documenting the effects of the COVID-19 crisis on the higher-education landscape, this study provides a snapshot of college student academic perceptions and emotional well-being at the end of May 2020. Roughly one-third of students perceived their academic future to be at high risk due to COVID-19. Similarly, about 30% of students indicated that they were likely to reduce or withdraw from classes in the Fall of 2020, should these classes be conducted online. Importantly, this study assessed students’ perceptions, not actual academic decisions (e.g., the decision to enroll in classes). However, the initial reports, as of January 2021, indicate that undergraduate enrollment across all the types of higher education institutions is down about 4% from the previous year; a decline that is twice the rate from the previous Fall 2019 enrollment (National Student Clearinghouse Research Center, 2020).

Consistent with previous research on emotional well-being in college students during COVID-19 (e.g., Ma et al., 2020; Son et al., 2020), a significant proportion (about one-third) of students reported difficulty coping with COVID-19 related disruptions and the elevated levels of stress. Given research showing that college students are at particularly high risk for adverse mental health outcomes (Son et al., 2020), this study demonstrates that these concerns likely persist and, in fact, may be exacerbated by the pandemic. Interestingly, students’ emotional well-being was significantly related to academic perceptions but was unrelated to perceptions of COVID-19. Likewise, perceptions of COVID-19 were related to each other (e.g., perceptions of disease severity correlated with frequency of engaging in risk management behaviors) but were unrelated to academic perceptions. Thus, students are experiencing the high levels of stress, difficulty coping with COVID-19 disruptions, and have academic concerns specific to COVID-19, yet these variables were unrelated to their perceptions of COVID-19 itself.

These results above suggest that emotional well-being may have a stronger relationship with variables that have a more “immediate” impact on students’ lives, rather than their overall perceptions of the disease itself. For example, academic performance or changes in the home environment (e.g., those imposed by social distancing/lockdown measures) may impact students’ well-being or academic beliefs more than perceptions of the virus. Indeed, students coping well with COVID-19 disruptions (a measure assessing the immediate impact of COVID-19) were more likely to agree that the transition to an online teaching format was the correct choice.

Of note, none of the participants in this sample reported testing positive for COVID-19, and the vast majority (97%) reported that no immediate family member tested positive. Therefore, the relationship between disease perception and emotional well-being should be tested in a sample that has more direct experience with the virus (e.g., changes in stress and coping before and after a positive diagnosis of COVID-19). Likewise, this survey investigated coping with COVID-19 disruptions *via* a single-item in order to understand how students’ perceptions of those disruptions impact their emotional well-being. It is important to acknowledge that students utilize different mechanisms for dealing with stress (coping strategies). For example, college women tend to use more emotion-focused coping strategies compared to college men (Brougham et al., 2009). Further, students’ lifestyle habits and coping strategies can effectively mitigate stress, but not all strategies are equally effective and different races/genders utilize different strategies (Welle and Graf, 2011). Thus, future investigations would benefit from a deeper investigation of which coping strategies may be particularly effective for students during the COVID-19 pandemic.

In line with previous research (Gunthert et al., 1999; Mak et al., 2010; Roddenberry and Renk, 2010; Widiger and Oltmanns, 2017; Kroencke et al., 2020), higher neuroticism and a more external locus of control were related to greater academic concerns and worse emotional well-being. This suggests that some students may be particularly at risk for poor emotional well-being during the pandemic. Exploratory analyses revealed that females in our sample reported higher stress levels and worse coping with COVID-19 disruptions than males. This gender difference in emotional well-being could be partly explained by the higher levels of neuroticism seen in our female sample as is typical of research on gender differences in personality (e.g., McCrae and Terracciano, 2005). It is also possible that female students face unique stressors during the pandemic that contribute to poor emotional health. For example, female students may be more likely to take on additional domestic or caregiving responsibilities during quarantine compared to male students. This seems a likely possibility, as previous research shows that females are disproportionately likely to serve as caregivers for ill family members compared to males (Bott et al., 2017). Balancing caregiving responsibilities with academic work may place female students at particular risk for negative mental health outcomes during COVID-19. Future research should investigate the role of both neuroticism and additional responsibilities faced by female students on their mental health.

Alarmingly, the students of color reported the perceptions of greater risk for their academic future and the higher likelihood of reducing or withdrawing from online classes in the Fall of 2020. In fact, according to recent surveys by the National Student Clearing House, Fall 2020 enrollment for minority students is down 6–10% from the previous year’s numbers (National Student Clearinghouse Research Center, 2020). This is in line with data showing that ethnic minority students are disproportionately likely to suffer negative educational consequences due to the pandemic (NAACP, 2020). These findings are particularly disconcerting, as they indicate that pre-existing inequalities in access to quality education are likely to continue to widen. Additionally, minority college students are more likely to rely on higher education institutions to meet basic needs, such as food and housing (NAACP, 2020), thus, withdrawal from classes during the pandemic has the potential to create problems beyond the interruption of education. Institutions of higher education should be cognizant of discrepancies in both academic and basic needs for minority students and work toward the implementation of interventions to support these students.

Though these data reveal several interesting relationships between academic perceptions, emotional well-being, and personality, they do not imply causation. The diversity of our sample (majority White and female) largely reflects the institution, where the survey was created and is not representative of all United States undergraduates. Additionally, our survey did not differentiate individuals from a socioeconomic perspective. It is likely that along with ethnicity, socio-economic inequities exacerbate pre-existing achievement gaps among students in higher education (Borman and Rachuba, 2001; Stephens et al., 2012). Indeed, it is possible that students’ perceptions and risk behaviors regarding COVID-19 do impact their academic perceptions and emotional well-being, but the relationship is moderated by factors related to socio-economic status (e.g., reliable internet access). Given the cross-sectional nature of the current study and limitations addressed above, longitudinal studies are needed to assess the long-term impact on student academic perceptions and emotional well-being.

## Data Availability Statement

The raw data supporting the conclusions of this article will be made available by the authors, without undue reservation.

## Ethics Statement

The studies involving human participants were reviewed and approved by the Arcadia University Institutional Review Board. The patients/participants provided their written informed consent to participate in this study.

## Author Contributions

AC, JD, and LF contributed significantly to the development, implementation, analysis, and subsequent reporting of this study. AC is the corresponding author (data available upon request). JD prepared all figures. LF assisted with editing and finalizing references. All authors contributed to the article and approved the submitted version.

### Conflict of Interest

The authors declare that the research was conducted in the absence of any commercial or financial relationships that could be construed as a potential conflict of interest.
